# *Actinostachysminuta*, a new species of grass fern from Mindanao, Philippines

**DOI:** 10.3897/phytokeys.151.53100

**Published:** 2020-06-12

**Authors:** Victor B. Amoroso, Fulgent P. Coritico, Peter W. Fritsch

**Affiliations:** 1 Center for Biodiversity Research and Extension in Mindanao (CEBREM), Central Mindanao University, Musuan, Bukidnon 8710, Philippines; 2 Department of Biology, College of Arts and Sciences, Central Mindanao University, Musuan, Bukidnon 8710, Philippines; 3 Botanical Research Institute of Texas, 1700 University Drive, Fort Worth, Texas 76107-3400, USA

**Keywords:** ferns, lowland tropical rainforest, Mount Hamiguitan Range Wildlife Sanctuary, Schizaeaceae

## Abstract

*Actinostachysminuta* Amoroso & Coritico (Schizaeaceae), from Mindanao, Philippines, is described herein as a new species. This species is distinguished from all other species of *Actinostachys* (grass ferns) by its notably short and narrow fronds, distinct triangular stipe, and bifid apex of the sorophore lamina with profuse white long hairs. This species is distinct from the other known Philippine species of *Actinostachys* by its diminutive epiphytic habit and a habitat restricted to the trunks of the tree fern *Sphaeropterispolypoda* (Baker) R.M.Tryon. A taxonomic key to the species of Philippine Schizaeaceae that incorporates the new species is provided.

## Introduction

The fern family Schizaeaceae comprises two genera, *Actinostachys* and *Schizaea*, and ca. 30‒35 species widely distributed in tropical and south-temperate regions (PPG I 2016; Smith et al. 2006; Chen et al. 2017). In the Philippines, the family is represented by two species of *Schizaea*: *S.dichotoma* (L.) Sm. and *S.malaccana* Bak., and two species of *Actinostachys*: *Actinostachysdigitata* (L.) Wall. and *A. inopinata* (Selling) C.F.Reed (Barcelona et al. 1996). Of these, *S.dichotoma* and *S.malaccana* are distinctive by having the sorophores attached pinnately to an elongate axis. *Schizaeadichotoma* is easily recognized by the dichotomously branching sterile portions of the frond, whereas *S.malaccana* has an unbranched sterile portion. The two species of *Actinostachys*, *A.digitata* and *A.inopinata*, both have digitately arranged sorophores and are differentiated by the relative width of their lamina and by the number of sporangial rows (4-seriate or biseriate, respectively) (Holttum 1955; Barcelona et al. 1996). Moreover, the different species of *Schizaea* and *Actinostachys* are classified into sub-genera and sections based on the exospore (smooth or striated to pitted) and spore size (Reed 1947).

All four Philippine species of the family Schizaeaceae are reported in Mount Hamiguitan Range Wildlife Sanctuary (MHRWS), a protected UNESCO World Heritage Site (Amoroso et al. 2016; Amoroso et al. 2018). During fieldwork in MHRWS in 2016 and 2018, we encountered unusual individuals of *Actinostachys* growing on the trunks of tree ferns. On careful examination of these plants, available type images from JSTOR Global Plants, and in consultation of the literature, we conclude that they represent a species new to science. Here we describe this new species and provide detailed photographs of it along with a key to the five Philippine species of Schizaeaceae, all of which are found in MHRWS.

## Taxonomy

### Actinostachys minuta

Taxon classificationPlantaeSchizaealesSchizaeaceae

Amoroso & Coritico sp. nov.

5B8ADE52-E2E8-5904-A5FF-15D8D33393AC

urn:lsid:ipni.org:names:77209910-1

Figures 1 , 2

#### Diagnosis.

This new species *Actinostachysminuta* is most similar to *Actinostachysplana* (Fourn.) Reed but differs by its shorter and narrow fronds with a distinct triangular stipe, sorophore lamina longer and narrower with white long hairs and sorophores 1‒4 but usually 1. It differs from the other four Philippine species by its restricted epiphytic habit on the trunk of the tree fern *Sphaeropterispolypoda* (Baker) R.M. Tryon.

#### Type.

Philippines • Mindanao Island. Davao Oriental: San Isidro Municipality, Mount Hamiguitan Range Wildlife Sanctuary, 622 m a.s.l., 10 October 2016, *V.B. Amoroso 11213* with F.P. Coritico (***holotype***: PNH; ***isotypes***: BRIT, CMUH).

#### Description.

Epiphytic on trunks of the tree fern *Sphaeropterispolypoda* with rhizome embedded between adventitious roots. ***Rhizome***: short-creeping to erect, black, becoming elongate, attached to the persistent tuberous gametophyte with profuse, long, uniseriate, pale brown hairs. ***Fronds***: crowded, pendulous, grass-like, unbranched, up to 3.0–4.5 cm long; ***stipe*** distinct, black, oblong to triangular in transection, 5–8 mm long, with short scattered glandular hairs, with a few large cortical sclerenchymatous cells, vascular tissues reduced with single flattened xylem strand; ***lamina*** (sterile portion) simple, unbranched, flattened, up to 2.5–3.7 cm × 0.8‒1.0 mm wide, margin entire, adaxial surface with distinct costa and scattered uniseriate hairs, the basal cells of the hairs persistent and forming scattered warts and disappearing distally; ***stomata*** arranged in one row (uniseriate) on each side of costa; ***sorophores*** 1 to 4 per frond but mostly 1, sessile or attached by a short stalk at apex of lamina, digitately arranged, 4–6 mm long; laminae of sorophores covered with profuse white long hairs adaxially, margin entire, apex bifid; ***sporangia*** in 2 rows, nearly symmetrically arranged, completely covering abaxial surface and protected by reflexed edge of sorophore lamina, sessile, ellipsoidal, with distal annulus, surface striated, glabrous; ***spores*** monolete, smooth.

**Figure 1.**
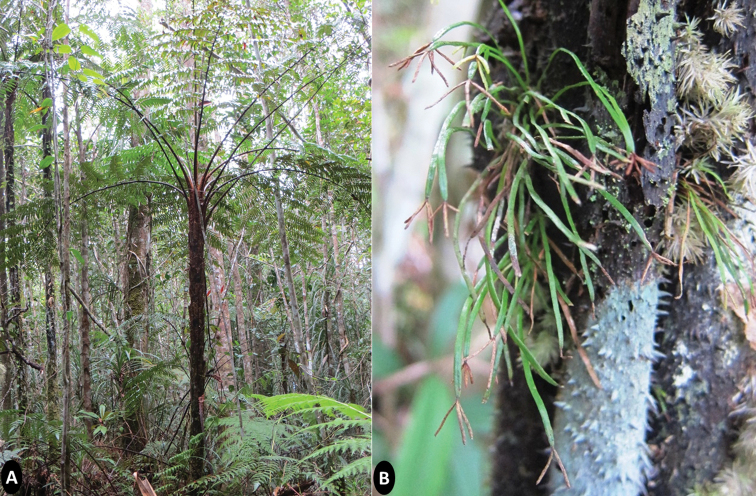
**A** the tree fern *Sphaeropterispolypoda* with associated vegetation. The trunks of this tree fern species serve as the substrate of *Actinostachysminuta***B** close-up view of *in-situ* epiphytic habitat of *A.minuta* embedded in the trunk of *S.polypoda* with the moss *Leucobryum* sp.

#### Distribution and habitat.

This species is currently known only within the buffer zone located outside the boundaries of MHRWS in San Isidro Municipality, in shaded habitat in lowland tropical rainforest at 622 m a.s.l. It grows strictly as an epiphyte on trunks of the tree fern *Sphaeropterispolypoda* with rhizomes embedded between adventitious roots in association with the moss genus *Leucobryum* and has not been observed terrestrially. The vegetation surrounding *Sphaeropterispolypoda* with *Actinostachysminuta* consists of trees 20–30 meters tall, including *Canariumasperum* Benth., *Dilleniaphilippinensis* Rolfe, *Gymnostomarumphianum* (Miq.) L.A.S.Johnson, *Lithocarpus* spp., *Pittosporumeuphlebium* Merr., and *Shoreapolysperma* Merr., and tree ferns such as *Alsophilalurida* (Blume) Hook. and *Sphaeropteriselmeri* R.M.Tryon. The ground cover is dominated by other fern and lycophyte species such as *Dicranopterislinearis* (Burm. f.) Underw., *Lindsaeagueriniana* (Gaudich.) Desv., *Nephrolepisbiserrata* (Sw.) Schott, *Schizaeadichotoma*, *Selaginellajagori* Warb., *Selligueataeniata* Parris, and *Taenitisblechnoides* (Willd.) Sw., as well as several species of *Calamus*.

#### Additional specimens examined.

Philippines, Mindanao, Davao Oriental Province, Municipality of San Isidro, Mt. Hamiguitan Range Wildlife Sanctuary, 06°44'15.24"N, 126°08'59.36"E, 622 m a.s.l., 16 June 2018, *V.B. Amoroso 13515* with F.P. Coritico (CMUH).

#### Etymology.

The specific epithet refers to the diminutive size of the fronds relative to the other species in the genus.

#### Suggested common name.

Diminutive grass fern.

#### Notes.

The traditional treatment of Schizaeaceae includes all species with digitately (connate) and pinnately arranged sorophores in *Schizaea* as in Barcelona et al. (1996), Barcelona (2011), Holttum (1955) and Kramer (1990). Here we follow the classification of PPG 1 (2016) segregating them into two genera *Schizaea* (species with pinnately arranged sorophores) and *Actinostachys* (species with digitately arranged sorophores) and by less easily observed features of their gametophytes (tuberous in *Actinostachys* versus filamentous in *Schizaea*) (Bierhorst 1968, 1971) and based on phylogenetic evidence (Labiak and Karol 2017; Wikstrom et al. 2002). The size of the fronds and sorophores is used in identifying the species of the family Schizaeaceae (Barcelona et al. 1996). However, the length of the sorophore is a much more reliable character than the length of the whole frond because the short sorophore is much less influenced by environmental conditions than the whole frond (Brownsey and Smith-Dodsworth 2000). In this respect, the new species and the closest related species (*A.plana*) differ from all other species of *Actinostachys* in its very short sorophores, about 2.5–6 mm long.

We compare the new species to three other digitate species of *Actinostachys* that are most similar to it in morphology based on the published descriptions of Bierhorst (1968), Holttum (1955), Barcelona et al. (1996), Reed (1947) and Sofiyanti et al. (2019), as well as examinations of JSTOR type images viz., *A.plana*, *A.spirophylla*, and *A.wagneri*. Our new species is closest morphologically to *A.plana*, followed by *A.spirophylla* Troll, and *A.wagneri* Sell. (Table 1). *Actinostachysminuta* shares an epiphytic habitat with *A.plana*, *A.spirophylla* and *A.wagneri*, growing in moss cushions on tree fern trunks, although Holttum (1955) did not explicitly mention tree fern trunks but simply trees.

**Table 1.** Major characters delineating *Actinostachysminuta* from *A.wagneri*, *A.spirophylla* and *A.plana*

**Character**	***A.wagneri* (Holttum 1955; Reed 1947)**	***A.spirophylla* (Holttum 1955; Reed 1947)**	***A.plana* (Reed 1947)**	***A.minuta***
***Habitat***	Epiphytic on stumps and bases of trees	Epiphytic in moss-cushions on trees	Epiphytic on tree ferns	Strictly epiphytic on trunks of the tree fern *Sphaeropterispolypoda*
Stipe	Indistinct	Indistinct	Distinct (5.5–7.5 long mm) flat	Distinct (5–8 mm long) triangular in transection
Lamina (sterile portion) length (cm)	6–20	4–8	5.5–10.2	2.5–3.7
Lamina (sterile portion) width (mm)	0.5–0.7	Ca. 1.5	1.4–1.74	0.8–1.0
Number of stomatal rows on each side of costa	1	1	Not described	1
Sorophore length (mm)	7–15	4–8	2.5–4.8	4–6
Sorophore number	2 to 5	1 to 3	3 to 6 but usually 4	1 to 4 but usually 1
Sorophore lamina apex	Not bifid, glabrous	Not described	Bifid or toothed	Bifid with profuse white long hairs
Sorophore lamina width (mm)	Not described	Not described	0.5–0.8	0.5–0.6 wide
Sporangia	Biseriate with brown hairs	Biseriate (sometimes apparently tetraseriate) and glabrous	Biseriate	Biseriate and glabrous
Spore surface	Striate	Striate	Smooth	Smooth
Distribution	Northeastern Australia, Indonesia (Borneo, Moluccas), Malaysia (Peninsular Malaysia), Papua New Guinea, Singapore, Thailand	Indonesia (Moluccas), Malaysia (Peninsular Malaysia), Micronesia (Ponape)	New Caledonia (Sommet du Mont Mi)	Philippines (Mindanao)

The sorophores of *A.plana* and *A.spirophylla* come closest in length to those of *A.minuta* (2.8–8 mm long versus 4–6 mm). In addition to the shorter sorophores, however, our new species is distinguished from *A.plana* by several other features, viz., shorter (2.5–3.7 cm long) and narrower (0.8–1.0 mm) lamina (sterile portion) with distinct triangular stipe up to 8 mm long (versus longer and wider (5.5–10.2 cm × 1.4–1.74 mm) and with flattened stipe in *A.plana*) and longer and narrower sorophores (4–6 × 0.5–0.6 mm vs. 2.5–4.8 × 0.5–0.8 mm).

**Figure 2.**
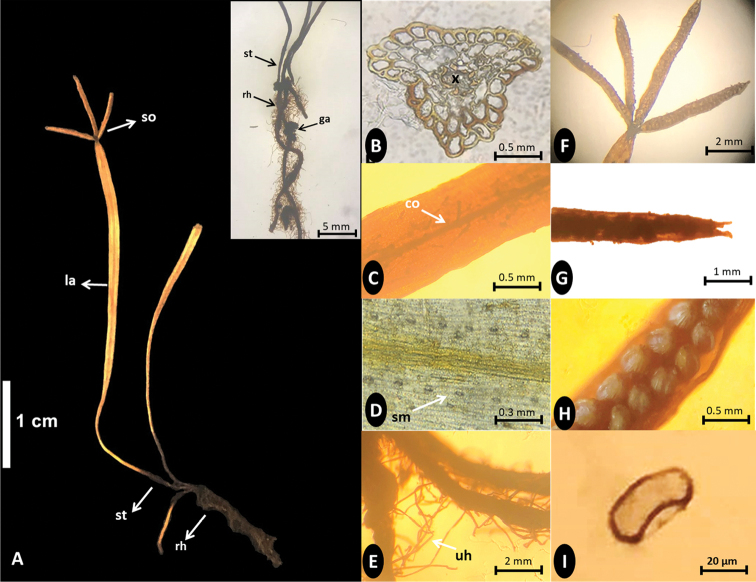
*Actinostachysminuta***A** habit with distinct stipe (st), unbranched fronds with 3 sorophores (so) attached at the tip of lamina (la), (inset) elongate rhizome (rh), with persistent gametophyte (ga) and uniseriate hairs **B** transection of the stipe showing flattened xylem (x) **C** lamina with distinct costa (co) **D** single row of stomata (sm) on each side of costa **E** elongated rhizome with long uniseriate hairs (uh) **F** digitate sorophores attached at the tip of the sterile lamina **G** bifid apical lamina of sorophore **H** portion of sorophore with reflexed lamina margins and biseriate sporangia, I. Monolete spore.

It is interesting to mention that we found a persistent gametophyte in our new species as also reported by Bierhorst (1968) in *Actinostachysoligostachys* Bierh., *A.melanesica* C.F.Reed, *A.intermedia* (Mett.) C.F.Reed, and *A.laevigata* (Mett.) C.F.Reed. The tuberous gametophyte is attached to the well-developed sporophyte (Fig. 2A).

All five species of Philippine Schizaeaceae are found in MHRWS in shaded forest. Except for *A.minuta* which is epiphytic on the trunks of tree ferns, the other four species are terrestrial, inhabiting an ultramafic soil with fallen leaves of *Gymnostomarumphianum*.

#### Conservation status.

Although MHRWS is a protected area, we only have observed this species from the type locality. The species occurs within the buffer zone of San Isidro, MHRWS with an estimated number of 30 individuals growing strictly on trunks of tree ferns. Its location in the buffer zone and the over-collection of tree fern trunks as a medium to grow other plants and for the making of handicrafts will likely reduce the populations of the species if this threat continues. Thus, we recommend listing the species as Critically Endangered (CR) based on its very small and restricted population with ≤ 50 mature individuals and the extent of occurrence estimated to be < 10 km^2^ (IUCN Standards and Petition Committee 2019).

### Key to the genera and species of grass ferns (Schizaeaceae) from the Philippines

**Table:** 

1a	Sorophores attached pinnately arranged or comb-like	***Schizaea***
2a	Fronds repeatedly dichotomously branched; lamina ≥ 2 mm wide	***S.dichotoma***
2b	Fronds unbranched; lamina < 2 mm wide	***S.malaccana***
1b	Sorophores digitate	***Actinostachys***
3a	Lamina ≥ 2 mm wide; sorophores 1.0‒5.0 cm long	**4**
4a	Lamina (sterile portion) ≤ 5 mm wide; stomata in one row on each side of costa; sporangia in four rows on the sorophores	***A.digitata***
4b	Lamina (sterile portion) ≤ 2.5 mm wide; stomata in two rows on each side of costa; sporangia in two rows on the sorophores	***A.inopinata***
3b	Lamina < 2 mm wide; sorophores < 1.0 cm long	***A.minuta***

## Supplementary Material

XML Treatment for Actinostachys minuta
